# Assessing the contribution of nonmarket factors to the market value generated by cow-calf operations in rangelands of the western USA: A true cost accounting approach

**DOI:** 10.1371/journal.pone.0296665

**Published:** 2024-05-31

**Authors:** Mauricio R. Bellon, Colleen M. Hanley, Becca B. R. Jablonski, Kevin Jablonski, Franklyn Garry, Ryan Rhodes, Mukunth Natarajan, Nicholas Benard, Kathleen Merrigan

**Affiliations:** 1 Swette Center for Sustainable Food Systems, Arizona State University, Tempe, Arizona, United States of America; 2 Maricopa County Cooperative Extension, Urban Agriculture Production, Small-Scale, and Beginning Farmer Program, University of Arizona, Phoenix, United States of America; 3 Department of Agricultural and Resource Economics, Colorado State University, Fort Collins, Colorado, United States of America; 4 Department of Animal Sciences, Colorado State University, Fort Collins, Colorado, United States of America; 5 Department of Clinical Sciences, Colorado State University, Fort Collins, Colorado, United States of America; UCL: University College London, UNITED KINGDOM

## Abstract

Food system transformation requires a better understanding of the negative and positive externalities involved in food production and consumption. Although negative externalities have received substantial attention, positive externalities have been largely overlooked. True Cost Accounting (TCA) is an economic assessment aimed at accounting for externalities in food systems. The beef industry is an important part of the US food system. In the western USA, beef cattle production is a major land use and economic activity that involves direct links among the cattle, range ecosystems, range management, climate, and ranchers’ decisions and welfare. We present a case study based on a TCA assessment to quantify and monetize the contribution of human, social, natural, and produced capitals, as well as farm structure, to the market value generated by cow-calf operations, a key component of the USA beef industry. We estimated an Ordinary Least Square regression model based on indicators of these capitals and of farm structure derived from publicly available data sources at the county level. From model coefficients, we estimated the marginal revenue product of these factors. Results show that nonmarket factors linked with human and social capitals support market performance by contributing to the market value of cow-calf production. These factors operate at scales above the ranch, usually remain hidden, and seldomly are considered in policy decision-making which can lead to policies that inadvertently hamper or eliminate these positive externalities.

## 1. Introduction

There is increasing recognition of the need to transform our food systems to be more sustainable and equitable [1–3]. An important part of this transformation requires a better understanding and quantification of the externalities involved in food production and consumption [2, 4–8]. An externality is “a positive or negative consequence of an economic activity or transaction that affects other parties without being reflected in the price of the goods or services transacted” [9 p.29]. Externalities can be positive or negative, happen at different scales, and vary depending on the type of food system involved. They not only are consequences of how food systems operate, e.g., carbon emissions from food production [10], but also can be crucial inputs to their operation, e.g., ecosystem services such as pollination, biodiversity [11] or nutrient cycling [12]. Externalities are not solely related to the natural environment but also involve important human and social dimensions [13]. The purpose of identifying and measuring externalities in a food system is to “internalize” them, i.e., to take them into account to make better decisions about the food we produce, consume, and support through public policies [4, 6, 14].

There is an expanding literature examining the negative externalities generated by food systems (e.g., [10, 15–17]). The Food Agriculture Organization (FAO) of the United Nations estimates that globally externalities of agrifood systems amount to at least USD 10 trillion annually [4], the magnitude of which underscores the importance of investigating externalities, which FAO refers to as hidden costs. For example, food systems globally account for 34% of total Greenhouse Gas (GHG) emissions, mostly from agriculture and land use change (71%) [10]. Food production produces ~ 32% of global terrestrial acidification and ~78% of eutrophication, mostly from the farm stage [15]. Beef cattle production is the largest anthropogenic source of methane emissions from enteric fermentation in the USA accounting for 194.9 MMT CO_2_e, representing 26.4% of total methane emissions and 3.1% of gross emissions in 2021 [18]. Poorly managed cattle grazing in rangelands can lead to land degradation [19], while cattle fencing can have deleterious effects on wildlife [20]. However, food systems also produce positive externalities, such as landscape and aesthetic value, water supply, nutrient fixation, soil formation, biodiversity, flood control and carbon sequestration, but they have not received as much attention [7]. Particularly, contributions of social factors outside the market have traditionally been ignored [8, 21, 22].

True Cost Accounting (TCA) is an emerging economic assessment aimed at making visible, measuring, and—if possible—monetizing critical positive and negative externalities in food systems [8, 14]. Externalities are assessed based on the reliance on, and effects of an agri-food value chain on four capitals: natural, produced, human and social. This assessment evaluates how these effects lead to changes in human well-being [9]. TCA was developed in recognition of the fact that just looking at yields, profits, calories, and/or proteins to judge the success and appropriateness of food systems is misleading and generates widespread unintended consequences and costs to the environment, human health, and equity [8]. TCA provides an approach for analyzing and valuing the contributions of nonmarket factors that create market value in an agri-food value chain. In its 2023 State of Food and Agriculture Report (SOFA) [4], FAO concludes that TCA should become the norm for decisionmakers seeking to direct global agrifood system transformation towards sustainability.

Beef production is an important agri-food value chain in the US food system [23–25]. The US beef cattle industry is generally divided into two sectors: cow-calf operations and cattle feeding [26]. Cow-calf operations consist of primarily range-fed cows that produce annual calves that, once weaned, are transferred to backgrounding/stocker feeding, feedlots, or kept on pasture and marketed directly by the ranch [27]. Cow-calf operations rely on grazing areas, which include grassland, pasture, range, and forest land, and constitute a key economic activity in the states located in the Intermountain Western region of the country, which according to the US Census Bureau [28] comprise Arizona, Colorado, Idaho, Montana, Nevada, New Mexico, Utah, and Wyoming (referred collectively as the West in the rest of the paper). Cattle grazing in this area takes place on almost 161.2 million hectares (73% of all land uses [29], involving 69,736 cattle operations with an inventory of 12.7 million head of cattle and sales of USD 10.3 billion in 2017 [30]. Cow-calf operations comprise direct links among the cattle, range ecosystems, range management, climate, and ranchers’ decisions and welfare.

Traditional analyses of the market performance of cow-calf operations have focused on production and financial factors at the operation/ranch level. These analyses include factors related to herd and range management, technology adoption, and cost structures of production, such as the costs of feed, invested capital, and paid labor (e.g., [31–36]). These analyses are based on the conventional metrics of yields and profits, however, for the most part ignoring the contributions of social capital, while also missing important aspects of human and natural capital for which there are no markets. In addition, they fail to account for structural factors that result from a broader array of assets that happen at the community level.

The aim of this paper is to quantify and monetize the contribution of factors outside the market, and beyond the conventional market metrics of yields and profits, to the market value of the cattle produced by cow-calf operations on rangelands of the West. We focus on indicators of human, social, natural, and produced capitals, as well as farm structure. In the language of TCA, we analyze and quantify the reliance of this market value with respect to the four capitals that underpin the TCA approach. We also investigate the role of farm structure as part of this process. *Farm structure* refers to the distribution of farm sizes and the inequality of farm landholdings [37, 38]. In the context of cattle production, since the key asset is not land but head of cattle, we interpreted *farm structure* as referring to the distribution of cattle operations (ranches) of different sizes (i.e., managing different number of head of cattle), while *inequality of holdings* refers to the level of concentration in holdings of head of cattle across operations of different sizes.

Our hypothesis is that nonmarket factors associated with human and social capital, as well as farm structure, make important contributions to the market value of cattle produced by cow-calf operations. To address our hypothesis, a statistical model was developed based on indicators of the four capitals that are the focus of TCA and of farm structure, derived from variables at the county-level for the West from the 2017 Census of Agriculture [39] and other publicly available data sources. Based on model results, a monetary value was estimated for the marginal contribution of each of these capitals and structural factors based on their association with the market value of the cattle produced by the cow-calf operations. Quantifying and monetizing the contributions of these factors should provide a more holistic view of the drivers of this agri-food value chain in the West, with the hope that unmasking these hidden relationships will aid to support better decision making in the future.

## 2. Background

### 2.1 Social capital

Social capital “encompasses networks, institutions, shared norms, values and understanding that facilitates cooperation among groups” [9 p.viii]. There is increasing evidence of the link between social capital and economic outcomes [40]. Social capital can contribute to improving production activities by reducing transaction costs, facilitating information flows, and improving market information [41]. The benefits of social capital include lowering the costs of negotiating collective actions and agreements, increasing the ability to exploit economies of scale, and increasing innovation by connecting farmer groups to research and extension organizations [7]. However, the contributions of social capital to agricultural production have been often overlooked [8]. Nevertheless, several studies have used social capital as a factor in a household production function showing its consistent positive and significant contribution to some measure of household welfare, such as income or expenditure, in agricultural settings [41–45]. For example, a recent study using a TCA approach [45], showed that social capital has a positive and significant influence on the value of production among smallholder farmers in villages that participated in the Andhra Pradesh Community Managed Natural Farming program in India. Other studies have used social capital as an input in a production function alongside other conventional inputs, such as land, labor, energy, fertilizers, or pesticides, to investigate the effect of social capital on production efficiency [22, 46, 47]. Results from several studies suggest that social capital can make a larger contribution to production than conventional inputs [22]. Social capital is not traded in markets and therefore there is no market price for it [47]. However, studies on production efficiency have been able to estimate a shadow price for social capital [22, 47]. In particular, Serra and Poli [22] showed that social capital was the second most valuable input after land among smallholder cotton farmers in India, supporting the hypothesis that social capital plays a significant role in an agricultural production process. Therefore, there is compelling evidence of the role of social capital on farmers’ welfare and on agricultural production. The studies reviewed above, however, have focused on the household level and have not examined the role of social capital at higher scales, though social capital is generated by interactions among households and communities. Social capital can be produced at a higher spatial scale, as Rupasingha et al. [48] have shown for counties in the USA. Furthermore, the evidence of the role of social capital in agricultural production is still limited and has focused mostly on smallholder farmers in the developing world, and as far as we know, it has not been investigated among cattle ranchers in the USA.

### 2.2 Human capital

Human capital refers to the knowledge, skills, competencies, and attributes embodied in individuals that facilitate the creation of personal, social, and economic well-being [9 p.viii]. Conventionally, human capital in the context of TCA encompasses issues of labor and working conditions, such as labor use and productivity, wages, underpayment, forced labor, labor rights, and workers’ health [9]. An important and measurable dimension of human capital is labor productivity, the amount of output produced per unit of labor invested. Labor productivity reflects the workers’ knowledge, skills, education, and experience, but also workers’ health and working conditions. In the USA, while agricultural output has tripled in the last fifty years, the quantity of labor used in the sector has decreased by nearly 76% indicating a major increase in labor productivity during the period [49]. This data is for the whole USA farm sector. No specific data on trends in labor productivity for cow-calf operations was found.

In the US agricultural sector, most operations are family businesses involving a substantial amount of self-employed and unpaid family labor, although hired labor also makes an important contribution to the sector [49]. While many family farms operate as business driven solely by profit-making considerations, others are driven by livelihood considerations that transcend profit-making [50]. In the latter case, the distinction between paid and unpaid labor is important because the contribution of unpaid labor to farm production, particularly to the extent it reflects family labor, may respond to a different set of incentives than for paid labor. While the use of paid labor should reflect its contribution to farm profitability, family labor could reflect contributions to family livelihood that involve considerations other than profit-making, and thus aspects outside market performance [50]. Reinhardt and Barlett [50] argue that a family farm uses a different accounting method than commercial operations to value their unpaid labor. For example, a family farm may not always consider alternative uses for their labor and the labor opportunity costs may not be subtracted from gross revenues. In fact, non-market interests such as lifestyle, identity, or family concerns among ranchers for their continuing involvement in cattle production have been well documented (e.g., [25, 51, 52]).

### 2.3 Natural capital

Natural capital refers to “the limited stocks of physical and biological resources found on earth, and of the limited capacity of ecosystems to provide ecosystem services” [9 p.viii]. In the case of rangelands where cow-calf operations take place, land area is the most basic quantitative indicator of the stocks of physical and biological resources and of the capacity of rangeland ecosystems to provide ecosystem services. However, land area does not consider variation in the types and qualities of the ecosystem services provided. Although rangelands supply multiple ecosystem services, such as forage production, recreation, and other nonspecified services, forage production is the most critical for livestock production [53]. As indicated by Jones et al. [54] the production of herbaceous aboveground biomass in rangelands is a fundamental ecosystem service upon which livestock, wildlife, and humans depend, but it is also influenced by human use and management. The type, amount, and quality of forage biomass production reflects the site potential (climate, soils, topography, and other abiotic factors) as well as the land use and management history of a given site. As such, forage biomass production can be seen as a factor that integrates multiple natural processes over time and space.

### 2.4 Produced capital

Produced capital refers to “all manufactured capital, such as buildings, machinery, physical infrastructure (roads, water systems), as well as all financial capital and intellectual capital (technology, software, patents, brands, etc.)” [9 p.viii]. This is the most straight forward capital to measure in monetary terms and most understood in society. In the case of cow-calf operations in the West, produced capital includes ranch infrastructure such as pumps and watering points, vehicles, fencing, vaccination equipment, and corrals. [55].

### 2.5 Farm structure

The scale and concentration of farms in the USA has increased substantially in the last thirty years, with much of agricultural production taking place among a small number of much larger farms [56]. Even though there is evidence of economies of scale in cow-calf operations [27], MacDonald et al. [56] find that the consolidation trend observed in other agricultural sectors has not happened, and cow-calf operations show little consolidation. Although there are concerns with the negative impacts of farm concentration, such as the impact on the viability of small farms and rural communities [56], the impacts of farm structure have not received much attention among cow-calf operations. In fact, there is evidence of positive impacts of a diverse structure of farms of different sizes. For example, interactions among different types of operations are important for the viability of small-scale operations, and the prevalence of smaller operations has positive spillover impacts to rural communities and economies [37, 57, 58]. Small operations can also benefit from being located near large operations because of positive spillover effects from the former to the latter, leading to positive welfare outcomes [37]. For example, large operations may attract private investment for commercial infrastructure that provide market opportunities for smaller operations in an area [59]. Medium and small operations may spend more of their profits locally fostering local business and creating larger multipliers effects in the local economy [37]. Therefore, a diversity of different types of operations can enhance infrastructure, investment, and market opportunities. Furthermore, there is evidence that the distribution of operation size matters for community well-being [57, 58]. For example, greater ratios of smaller-to-larger operations in non-metropolitan US counties were found to be associated with better community outcomes such as higher earnings per job, lower poverty rates, higher homeownership, lower financial stress, and better health outcomes; all factors associated with human and social capital [58]. Therefore, there is economic and sociological evidence that farm structure is associated with capital endowments in rural settings. As posited by TCA, ignoring these factors may lead to partial and biased assessments of the drivers of cattle production that are at the core of the beef cattle industry in the West and its environmental and social impacts.

## 3. Data and model

To operationalize the four capitals and farm structure we identified a set of variables in the 2017 Census of Agriculture [39] and other publicly available data sources. (**S1 Data contains all the data used in the paper**). For each of the variables, we provide links to the underlying tables obtained from the Quick Stats site of the US Department of Agriculture (USDA) National Agricultural Statistics Service (NASS) website (https://quickstats.nass.usda.gov/). All variables are at the county level. These variables were used as indicators of each of the capitals. Some of the variables had to be adjusted to make them more relevant for context of our study and we provide an explanation of the adjustments, as well as the limitations of the data.

To test our hypothesis, we estimated the following model:

$$Log\;Y_{i}=\beta C_{i}+\gamma S_{i}+\theta V_{i}+\varepsilon_{i}$$ (1)

Where Log Y_i_ is the natural logarithm of sales of cattle, including calves in US Dollars (USD) for county i; **C** is a vector of indicators of the four capitals; **S** is a vector of indicators of farm structure; **V** is a vector of covariates; and ε is the error term. The model is inspired by other studies that have quantified the contribution of social capital to some measure of welfare by using a reduce-form production function (e.g., [41–43]). These studies have used indicators of household welfare at the household level as dependent variable. Here a dependent variable was used at the county level, thus an aggregate across production units. A log linear specification rather than a log-log specification, the classical Cobb-Douglas production function specification, was used because several of the independent variables are expressed in terms of diversity indices and scores derived from a Principal Component Analysis (PCA) and it is not clear the appropriateness of expressing these types of variables as logs.

To estimate Eq 1, an Ordinary Least Squares (OLS) regression model was used. The model was estimated with the function “*lm_robust”* (with standard error type HC2) of the package *estimatr* version 0.30.2 in R [60]. The dependent variable is the natural logarithm of the sales of cattle, including calves, in USD in 2017 by county [30]. For some counties, no data was reported due to disclosure restrictions, while no cattle were produced in a few others. There was complete data for 235 counties, accounting for 84.5% of counties with at least one cattle operation and 87% of the total sales in the West. The variables used in the regression model and their sources are presented below.

For produced capital, two variables were used: (1) a weighted measure of the asset value of buildings, land, and machinery in USD as reported in the Census of Agriculture [61, 62], and (2) the inverse of the population-weighted mean distance in km to the nearest interstate highway on-ramp or intersection in 2007 as a measure of the road infrastructure available [21, 63]. (S1 Text in S1 File presents an explanation for the rationale and procedure used for the weighting of the asset value of buildings, land, and machinery).

For each county in the West two indicators of the natural capital relevant for livestock production were used: (1) the log of the number of acres in pastureland and pasture woodland as reported by the Census of Agriculture [62]; and (2) the first two principal components of a PCA of the average and coefficient of variation of the year on year biomass production of Annual Forbs and Grasses (AFG) and of Perennial Forbs and Grasses (PFG) in pounds per acre (ppa). The data was obtained from the Rangeland Production Dataset [64] for the period 1986 to 2017. The first two principal components had eigenvalues > 1. For ease of interpretation, the principal component scores were made positive by adding the absolute value of the score with the lowest negative value to all the other scores. Therefore, all scores were shifted accordingly, and the lowest score became zero. They accounted for 43.2% and 26.9% of the variance respectively (for a total of 70.1%) and reflect the long-term biomass productivity of the rangelands and its variation. The first principal component is highly and positively correlated with average PFG (σ = 0.70, p < .000) and to a lesser extent with average AFG (σ = 0.54, p < .000), and negatively with the coefficients of variation of AFG (σ = -0.73, p < .000) and PFG (σ = -0.64, p < .000). The second principal component is highly and positively correlated with AFG (σ = 0.77, p < .000) and the coefficient of variation of PFG (σ = 0.67, p < .000). (S2 Text in S1 File presents an explanation of the rationale for the use of a PCA variables used and the choice of variables).

The Rupasingha, Goetz, and Freshwater (RGF) Index was used as an indicator of social capital [48]. The index is the score of the first principal component derived from a PCA of the number of different types of organizations per capita, the percentage of voters who voted in presidential elections, the county-level response rate to the Census Bureau’s decennial census, and the number of tax-exempt non-profit organizations per capita, by county.

Four variables reported in the Census of Agriculture as indicators of human capital were used: (1) the average number of paid workers, (2) the average number of unpaid workers [65], (3) the average number of years of the primary producer on any operation [66] (a primary producer is “the person who made the most decisions for the farm” [67 p.B20], and (4) the age structure of primary producers in a county. As an indicator of age structure, a Simpson Diversity Index was used based on the ages of primary producers in a county calculated on the share of six age groups reported in the Census of Agriculture (i.e., 25–35; 35–44; 45–54; 55–64; 65–74; ≥75) [68]. This index is for primary producers of all operations, not just cattle operations since the publicly available Census data cannot be filtered accordingly. The index (A_i_) was calculated as follows:

$$A_{j}=1‐\sum {a_{j}}^{2}$$ (2)

where: a_j_ = number of primary producers in age group j/total number of operations; j = 1 to 6.

The average amount of paid labor reflects the stock of market labor available, while the average amount of unpaid labor may reflect the stock of family labor available since there is a strong correlation between the number of unpaid workers and persons in the household of primary producers (σ = 0.96, p < .000, S1 Fig in S1 File). We use the average number of workers for both categories to reduce potential biases because the Census reports the total numbers for all farming activities and not specific for ranching (as we used the publicly available data and not the restricted access data for our analysis). Furthermore, in the case of unpaid labor, there was a very high correlation between the number of unpaid workers and the number of operations, so to avoid multicollinearity the average number instead was used. The average number of years on any operation reflects farm or ranch work experience of the primary producer. Age structure was included due to concerns that the rural population is aging and about the impact of this process on agricultural production [69, 70], as well as of succession pathways from older to younger ranchers [71].

As an indicator of the distribution of cattle operations (ranches) of different sizes (i.e., managing different number of head of cattle) a Simpson Diversity Index was calculated on the share of operations (ranches) grouped into seven categories by the number of head of cattle per operation as reported in the Census of Agriculture (i.e., 1–9; 10–19; 20–49; 50–99; 100–199; 200–499; ≥ 500) [72]. The index (D_i_) was calculated as follows:

$$D_{i}=1‐\sum {o_{i}}^{2}$$ (3)

where: o_i_ = number of operations of category i/total number of operations; i = 1 to 7.

This indicator captures the interactions of cattle operations of different sizes that may generate spillovers. Following the approach of Chamberlin and Jayne [37], as an indicator of the level of concentration in holdings of head of cattle across operations of different sizes, the Gini Coefficient was calculated on the distribution of the number of head of cattle by the size of operations in a county [72]. A high Gini coefficient indicates a high concentration of head of cattle among a small proportion of operations.

The number of operations in a county was included as a covariate to correct for the fact that counties with more operations may have larger inventories and higher sales [72]. Since the focus is on cow-calf operations in rangelands, but the gross value of the sales of cattle and calves includes animals in dairy operations and cattle on feed, two additional covariates were included to account for their impact on the dependent variable: the share of operations with milk cows and the share of operations with cattle on feed relative to the total number of operations with cattle calculated from data of the Census of Agriculture [73].

## 4. Monetary contribution

To monetize the contribution of the indicators associated with the different capitals, the elasticities for each of the indicator variables were calculated for the four capitals and farm structure with respect to the gross value of the sale of cows and calves for each county. An elasticity represents the percentage change in the dependent variable for a 1% change in an independent variable. Elasticities are unitless so the contributions of different variables can be compared, which is particularly useful for indices of diversity, or scores derived from a PCA. Based on those elasticities, the marginal monetary value of their contribution was estimated (S3 Text in S1 File presents a more detailed explanation of the procedure used).

## 5. Results

Table 1 presents a summary of the mean and standard deviation per county of the variables used in the regression model. The total gross value of sales of cows and calves, the indicator of market value, of the 235 counties included in the regression in 2017 was USD 9,003,568,000, while the mean value per county was USD 37,830,118.

**Table 1:** Summary statistics per county of the variables used in the regression model^a^.

Variable	Mean	Std. Dev.
Log of the sales of cattle, including calves (Million USD)	16.52	1.44
Value of assets building, land, machinery (Million USD)^b^	192.61	147.02
Highway density^c^	0.94	1.17
Log of area in pastureland and pasture woodland	12.38	1.65
Principal Component 1- Annual biomass production	7.12	1.31
Principal Component 2- Annual biomass production	1.35	1.04
RGF Index of social capital	2.23	1.39
Average number of paid workers/operation	3.98	2.48
Average number of unpaid workers/operation	2.35	0.45
Years of experience of primary producer	24.09	3.26
Diversity in ages of primary producers	0.78	0.03
Diversity of operations of different sizes	0.77	0.09
Gini coefficient	0.71	0.12
Number of operations with cattle	250.85	237.07
Share of cattle on feed	0.01	0.02
Share of milk cows	0.05	0.07

^a^ N = 235 counties

^b^ This variable was weighted by the ratio of the Census variable “cattle, incl calves–operations with inventory” to the variable “ag land, incl buildings—operations with asset value” per county.

^c^ Inverse of the population-weighted mean distance in km to the nearest interstate highway on-ramp or intersection.

Heteroscedasticity was tested with the Breusch-Pagan / Cook-Weisberg test and the null hypothesis of constant variance was rejected (p < .004). To correct for this issue, White’s robust standard errors in the regression estimation were used. The variance inflation factor (VIF) test was applied, and no evidence of multicollinearity was found. Spatial autocorrelation in model residuals was tested with Moran’s I test and no evidence for it was found (p = 0.158).

Regression results (Table 2) showed that the model worked well, explaining 82.4% of the variance. Except for primary producers’ years of experience, highway infrastructure, and share of milk cows, all other indicators of the different capitals were statistically significant, indicating their relevant contribution to the market value of cow-calf operations.

**Table 2:** Regression results with robust standard errors^a^. Dependent variable: Natural log of the gross value of the sales of cattle and calves 2017 (USD).

Variable	Coefficient	Std. Error	t-value	Signif.
Intercept	0.175	2.170	0.081	
Value of assets building, land, machinery	0.003	0.001	5.445	***
Highway density	-0.042	0.038	-1.082	
Log of area in pastureland and pasture woodland	0.219	0.051	4.296	***
Principal Component 1-Annual biomass production	0.136	0.035	3.890	***
Principal Component 2-Annual biomass production	0.126	0.053	2.361	**
RGF Index of social capital	0.108	0.044	2.471	**
Average number of paid workers/operation	0.084	0.037	2.262	*
Average number of unpaid workers/operation	-0.264	0.117	-2.254	*
Years of experience primary producer	0.004	0.024	0.183	
Diversity in ages of primary producers	7.259	2.819	2.575	**
Diversity of operations of different sizes	5.934	0.715	8.297	***
Gini coefficient	1.923	0.682	2.821	***
Number of operations with cattle	0.001	0.000	2.178	*
Share of cattle on feed	11.540	3.263	3.537	***
Share of milk cows	-0.890	1.394	-0.638	

^a^ N = 235. Significance at the .05; .01; .001 level indicated by

*. **, *** respectively for a two-tail t-test. Adjusted R-squared: 0.824, F-statistic: 58.18 on 15 and 219 DF, p-value: < 2.2e-16.

The value of buildings, land, and machinery, an indicator of produced capital, was highly and positively associated with the market value of cow-calf production. In the case of natural capital, the three indicators used were positively and significantly associated with market value. Results show that the larger the area of pastureland and pasture woodland in a county the more market value the cow-calf production generates since more animals can be maintained. The positive association of the first principal component, which captured most of the variation in the average long-term productivity of the range and its variation in a county, indicate that cow-calf production in counties with higher average long-term annual biomass production of PFG and AFG and lower variability in both of these variables generate higher value. The second principal component which captured slightly above half of the variation captured by the first component was also positive and statistically significant. The positive contribution of social capital to the market performance in cow-calf operations is supported by the positive and significant association with the RGF Index of social capital. The results from human capital should be interpreted carefully since the indicator variables used refer to all agricultural operations, not only to cow-calf operations. However, since the associations are with cattle production, it is assumed that these variables can capture, at least partially, specific associations between human capital and cow-calf production. Results show that the average number of paid workers in a county was positively associated with the market value of cattle sales. However, the negative association of unpaid labor suggests that counties with a higher average number of unpaid workers generate a lower market value from cattle sales. The diversity of cattle operations of different sizes was positive and statistically significant. The Gini coefficient had a positive and highly significant association with market value, indicating that counties where the majority of the cattle inventory are concentrated on a few large ranches generate higher gross sales value.

In terms of the monetary value of the marginal contributions of these variables per county, results show that the diversity in ages of primary producer was the largest value, followed by the diversity of operations of different sizes, and then the Gini coefficient, all structural factors. Among the indicators of the four capitals, the principal components of annual biomass production associated with natural capital together had the highest value, followed by the value of assets building, land, and machinery, indicator of produced capital. Then the value of the RGF Index, indicator of social capital. The lowest values were associated with indicators of human capital, with paid labor with the lowest positive value, and unpaid labor with a negative value (Table 3).

**Table 3:** Elasticities of indicator variables of the four capitals and monetized average value of their marginal contributions per county.

Variable	Elasticity	Marginal value of contribution (USD)
Value of assets building, land, machinery	0.57	219,313
Area in pastureland and pasture woodland	0.22	84,171
Principal Component 1-Annual biomass production	0.98	375,831
Principal Component 2-Annual biomass production	0.16	61,872
RGF index of social capital	0.24	91,537
Average number of paid workers/operation	0.33	125,885
Average number of unpaid workers/operation	-0.63	(242,184)
Diversity in ages of primary producers	5.69	2,177,964
Diversity of operations of different sizes	4.61	1,763,873
Gini coefficient	1.35	517,536

## 6. Discussion

Results show that the indicators of the four capitals and farm structure make important contributions to the market performance of cow-calf operations. In particular, they support our hypothesis that factors associated with human and social capitals and farm structure outside the market contribute to the market value generated by cow-calf operations in the Western USA. We found a positive and significant association of social capital, which is consistent with the findings of other studies [22, 41–45]. Our study, however, shows that this contribution can take place at scales above the household.

The large contribution of the diversity of operations of different sizes is noteworthy and suggests that the interactions among cattle operations of different sizes may contribute to the creation of multiple positive spillovers, e.g., business opportunities for supporting goods and services, job opportunities, as well as smaller operations benefiting from the services that the presence of larger operations enable (e.g., [23, 57, 58]). Some of these spillovers have also been associated with social capital, such a reducing the costs of conducting business and increased capacity to innovate [7]. Although these interactions reflect farm structure, they involve social capital to the extent that aspects of social capital enable the interactions among heterogenous groups to take place [74]. These positive spillovers could be seen as a Marshallian externality, i.e., spillover effects external to the firm but internal to the sector, such as the pool of specialized labor and suppliers, and intra-industry knowledge [75]. Another possible benefit of a greater diversity in the size of cattle operations may be that it enables a more efficient exploitation of the range of biophysical conditions in a county. Unfortunately, there was no data to address this explanation. This is an area for further research.

Regarding human capital, the positive and highly significant association of the diversity in ages of primary producers, indicating that as the diversity of producer ages increases so does the value of cattle sales, suggests that the interactions among producers with different levels of experience and capacities contribute to generating positive market value. Furthermore, it suggests the importance of fostering human capital and implies an important role for existing operations to play in training the next generation as the rural population ages [69, 70] and for nurturing succession pathways from older to younger ranchers [71]. Consequently, age structure matters to the present and future of human capital in ranching and the long-term viability of this industry.

Another important result related to human capital is the negative association of unpaid labor with the value of cattle sales. This result is consistent with the view that unpaid labor mainly involves family labor because as Reinhardt and Barlett [50] have argued, family labor responds to a different set of incentives than paid labor, reflecting contributions to family livelihood that involve considerations other than profit-making, and are subject to a different accounting method than commercial operations. These non-market incentives and willingness to accept lower returns on investment in ranching because of non-market interests such as lifestyle, identity, or family concerns, are well documented in the literature (e.g., [25, 51, 52]. Therefore, the loss in market value associated with unpaid labor can be seen as an implied subsidy that ranchers provide to society due to the cultural and personal benefits ranching generates. In other words, the socio-cultural impact of ranching can be valued at an average of USD 242,964 per county (the decreased cow-calf market value associated with unpaid labor). This amount is not insignificant compared with the values associated with the other capitals. This study suggests the value of further exploring why many cow-calf operations persist, despite low- to no-profitability, and how these entities contribute to the viability of rural places.

The positive association of the Gini coefficient and the market value of cattle sales is consistent with the idea that consolidation of operations is associated with higher profitability of cattle operations [27]. Contrary to the report that cow calf operations have not been a part of the consolidation trend observed in other agricultural sectors [56], our results find that less than 7% of operations across the West, those with 500 head or more, accounted for almost 70% of all head of cattle, while operations with less than 10 head accounted for 34% of operations but less than 1% of all head of cattle, which translates into an overall Gini coefficient of 0.8, indicating that there is a great deal of concentration among ranches in the West.

Diversity of operations of different sizes and the Gini coefficient of the distribution of the number of cattle by the size of operations, variables associated with farm structure, make a large and positive contribution in terms of size, statistical significance, and estimated monetary values. These results may initially seem contradictory (concentration versus diversity). Under further analysis, however, these results suggest that the way farm structure influences the market value of cow-calf operations is complex and operates through different mechanisms beyond the level of the ranch. Farm structure is a multidimensional concept [37], and these results may be reflecting this multi-dimensionality and suggest that there may be a trade-off between the benefits and costs of a diversified versus a concentrated farm structure in cattle ranching. This is another area that merits further research to understand the underlying mechanisms and trade-offs. For example, greater understanding will aid USDA as it invests more resources in meat processing capacity and builds out infrastructure and business support to boost local agriculture entrepreneurship [76].

The positive and significant association of the three indicators of natural capital confirm the crucial role of this capital for cow calf operations. The two principal components associated with different parts of annual biomass production were both significant. This result suggests that even after accounting for the impact of the conditions captured by the first principal component, counties with higher average AFG long-term production, but with higher PFG variance were associated with higher market value. Interpreting these results requires a further understanding of the natural and anthropogenic management factors affecting plant species composition and resulting diversity. The benefits of greater plant diversity and compositional heterogeneity remains an active area of research in range science [77].

The indicator of produced capital is positively and significantly associated with cattle sales which is not surprising since it reflects a capital that is fully accounted in the market. The value of its inclusion is that ensures that the magnitude and significance of the contributions of the nonmarket factors on cattle sales have been corrected for the capital that has a market value.

Taken together, the diversity in ages of primary producers and the diversity of operations of different sizes suggest that interactions among producers and their operations generate positive spillovers at the county level. Understanding how these interactions create market value is an important area of research. For example, studying how producers of different ages in an area actually interact and the resulting effects on knowledge-sharing, risk management, and resource-sharing. As indicated these interactions may be a form of social capital. Additionally, investigating whether interactions among ranches of different size in an area create business opportunities, a better supply of services, more job opportunities, or other forms of positive social interactions merit further research to confirm that in fact this diversity creates a richer social environment, and as such can be seen as part of social capital that is conducive to more successful ranching operations. It is interesting to note that both the USDA Climate Smart Commodities Program and the Transition to Organic Partnership Program facilitate mentor-mentee farmer pairings as a strategy to accomplish program goals; there may be things to learn from the social capital built among cow-calf communities that can inform other dimensions of USDA work [78, 79].

A key contribution of this research is to show the importance of considering farm structure in assessing externalities in food systems by providing evidence of the role of nonmarket factors that operate at scales above the ranch in creating market value for beef cattle production and estimating monetary values for them. However, the monetization of these nonmarket factors can be controversial. This is an area of contention in the TCA literature (e.g., [8, 80]. The point of monetization in TCA is not to commoditize or privatize nature or other nonmarket aspects of food systems, but to make the system more transparent in order to facilitate more informed decisions and better incorporate externalities [8]. It is more intuitive to interpret a dollar amount than a regression coefficient or an elasticity. The most useful results may not be the absolute monetary values, but their relative magnitudes and their ranking that could be used to prioritize the research issues mentioned above and potential areas of policy intervention. Therefore, at a minimum, monetizing these contributions can serve as a heuristic device to better support decision making.

This study has several limitations. The study is observational, and the empirical methodology applied here only allowed to test for associations that were interpreted as indicators of the underlying causal relationships present in the system analyzed here. This is an issue common to most TCA studies since they typically rely on observational data. The variables used as indicators of the different capitals were limited by the use of publicly available data. Much of this data is not specific to cow-calf operations, but refer to all to all agricultural operations, adding uncertainty to the interpretation of the results. However, the benefit of our use of county-level publicly available indicators is that it allows for replicability. The loglinear specification of the statistical model assumes perfect separability and additivity of the four capitals and the structural factors examined, which is likely not completely accurate. We were constrained by the way we operationalized some of the variables, such as principal component scores and diversity indices where their transformation to logs for an alternative specification may not have been appropriate. The estimated monetary values reported are averages across counties and do not capture the considerable variation that exists among them, but they do provide a general idea of the magnitudes and relative importance of the contribution of these factors. In spite of these limitations, model results have high statistical significance and provide important insights and information about the contribution of factors that are not usually considered when analyzing the market performance of cow-calf operations.

## 7. Policy implications

Policymakers almost always face tradeoffs when designing policy and thus need to understand how actions in one area may affect outcomes in another [4]. For example, the TCA of cow-calf operations in the western USA suggests that the cost of externalities associated with natural capital varied between USD 2.92 and 48.26/kg of Carcass Weight (CW) depending on the rate of soil carbon sequestration, compared to a market price of USD 3.25/kg of CW, which may dissuade policymakers from continued support of the sector, particularly with mounting and real concerns over cattle’s contributions to GHG emissions [55]. Yet, the cow-calf segment of the beef livestock value chain is vast; it is the primary user of western land and supports nearly 70,000 operations. With concerns about the “hollowing out of rural America,” a phrase describing the outmigration of young people from rural communities, policymakers may decide that it is important to support cow-calf operations as part of government investments aimed at improving the quality of life in rural areas [81]. All told, issues related to beef livestock production, such as farm structure and consolidation, climate smart production practices, and healthy diets–are the subject of debate among USA policymakers writing farm bill legislation [81, 82]. In particular, this study shows how valuable nonmarket factors can be for the market performance of cow calf operations. However, these factors are hidden and usually not considered in policy decision-making which can lead to policies that inadvertently hamper or eliminate these positive externalities. For example, the great emphasis on land consolidation and achieving greater economies of scale among agricultural operations [56], may eliminate the substantial benefits of maintaining a diversity of cattle operations of different sizes that we have documented here. This study contributes to greater understanding of the full costs and benefits of cow-calf operations by considering externalities and in doing so, aids efforts to anticipate the impact of enacting various policy proposals.

## 8. Conclusions

Based on a TCA approach that aims to make hidden factors visible to make better decisions in food systems, our results show the importance of accounting for non-market aspects of human and social capital, as well as spillover effects from the interactions among a diversity of ranches and ranchers when analyzing the market performance of cow-calf operations. This is not implying that the conventional metrics of yields and profits at the ranch level are wrong, but that they are incomplete. Results suggest that by ignoring these factors, crucial aspects of these operations may be missed. There is a need for a better understanding of how these factors operate to inform policies that enhance the benefits that ranchers involved in the cow-calf operations in the West derive from human and social capital and from interactions among themselves and with society.

## Supporting information

S1 Data Data nonmarket factors. (ZIP)

S1 File (DOCX)
