# Supplementary material for: Assessing the contribution of nonmarket factors to the market value generated by cow-calf operations in rangelands of the western USA: A true cost accounting approach
Source: PLoS One. 2024 May 31;19(5):e0296665. doi: 10.1371/journal.pone.0296665 (PMC11142599; doi:10.1371/journal.pone.0296665)
Supplement: S1 File — (DOCX) [file pone.0296665.s002.docx]

# Supporting Information

## S1 Text. Produced capital.

The asset value of buildings, land, and machinery in the Census refers to all farming operations, not just operations with cattle and calves. Therefore, to make this variable more specific to the modelling context of our study, it was weighted by the ratio of the Census variable “cattle, incl calves – operations with inventory” to the variable “ag land, incl buildings—operations with asset value” per county.

## S2 Text. Natural capital

As indicated earlier, land area is the most basic quantitative indicator of the stocks of physical and biological resources and of the capacity of rangeland ecosystems to provide ecosystem services. Although in theory, this should be reflected in the land market value already included in the asset value of buildings, machinery, and land used as the indicator of produced capital, in reality this may not be the case. A large amount of the grazing lands in the West are public and the federal grazing fee is set by the federal government, not from a competitive market, thus the forage value of a significant part of these lands can be seen as a nonmarket good (Maher et al., 2021, p. 4). Therefore, the asset value of the land included in the measure of produced capital fails to capture its true value and justifies its inclusion as part of the natural capital directly. Since land area does not consider variation in the types and qualities of the ecosystem services provided, we capture these qualities through yearly measurements of annual and perennial forbs and grass biomass production (AFG and PFG). These estimates were obtained from the Rangeland Production Dataset (Rangeland Analysis Platform, 2022) for the period 1986 to 2017. The average and coefficient of variation of AFG and of PFG were calculated for the period by county. A PCA was carried out on the averages and coefficients of variation of AFG and PFG. Through the use of a PCA, we are able to capture the potential interactions among the average values and their variability across time.

## S3 Text. Monetary contribution

The elasticity of each indicator variable was calculated by multiplying the respective coefficient from the estimated regression model of Eq. 1 by the value of the indicator variable of a county. To obtain the marginal monetary value of the contribution of a variable to the market value of cattle production, the respective elasticity was divided by 100 and then multiplied by the value of the gross sales of cattle of the county. Reported are the average values of the elasticities and the monetary values across all the counties included in the regression.


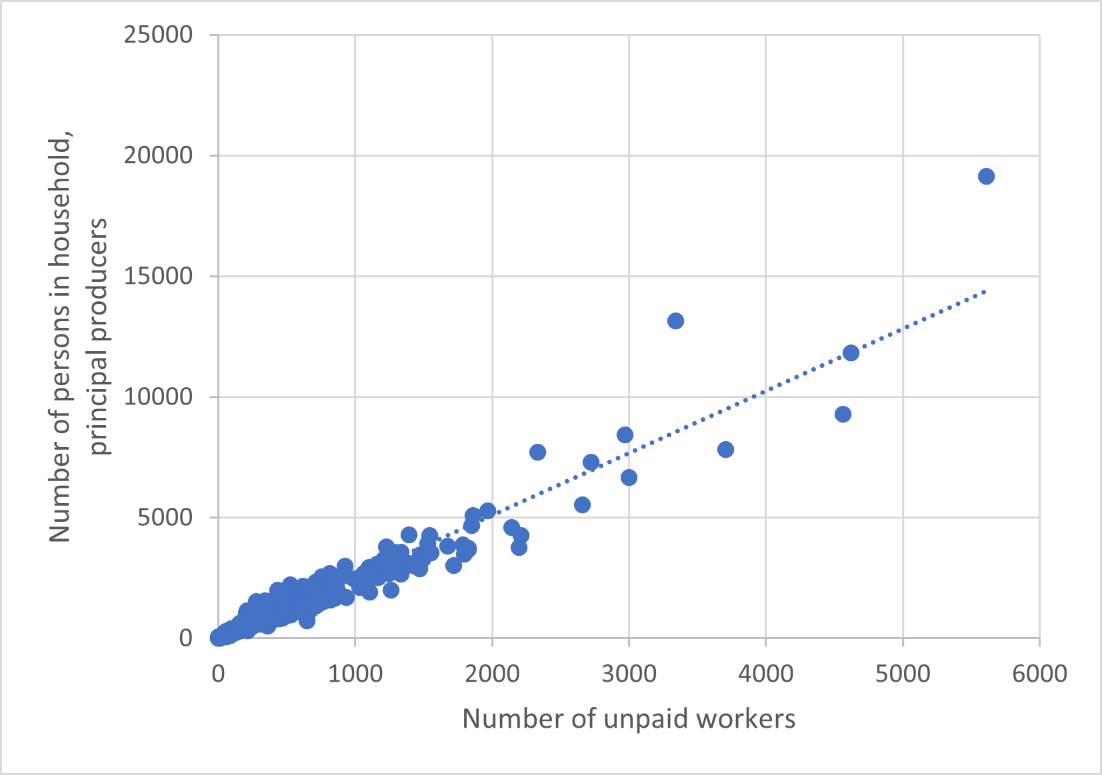


Fig S1. Relationship between the number of unpaid workers and the number of persons in the households of principal producers.

References

Maher AT, Quintana Ashwell NE, Maczko KA, Taylor DT, Tanaka JA, Reeves MC. An economic valuation of federal and private grazing land ecosystem services supported by beef cattle ranching in the United States. Translational Animal Science. 2021 Jul 1;5(3):txab054.

Rangeland Analysis Platform. Rangeland Production Dataset. January 27, 2022. Available from <https://support.rangelands.app/article/49-rangeland-production>
